# Differences in the Structure and Antimicrobial Activity of Hydrazones Derived from Methyl 4-Phenylpicolinimidate

**DOI:** 10.3390/ma15093085

**Published:** 2022-04-24

**Authors:** Katarzyna Gobis, Małgorzata Szczesio, Andrzej Olczak, Izabela Korona-Głowniak, Ewa Augustynowicz-Kopeć, Ida Mazernt-Politowicz, Dagmara Ziembicka, Marek L. Główka

**Affiliations:** 1 Department of Organic Chemistry, Medical University of Gdańsk, 107 Gen. Hallera Av., 80-438 Gdansk, Poland; dagmara.ziembicka@gumed.edu.pl; 2 Institute of General and Ecological Chemistry, Faculty of Chemistry, Lodz University of Technology, Zeromskiego 116, 90-924 Lodz, Poland; malgorzata.szczesio@p.lodz.pl (M.S.); andrzej.olczak@p.lodz.pl (A.O.); ida.mazerant-politowicz@dokt.p.lodz.pl (I.M.-P.); marek.glowka@p.lodz.pl (M.L.G.); 3 Department of Pharmaceutical Microbiology, Faculty of Pharmacy, Medical University of Lublin, 1 Chodźki Str., 20-093 Lublin, Poland; iza.glowniak@umlub.pl; 4 Department of Microbiology, National Tuberculosis and Lung Diseases Research Institute, 26 Płocka Str., 01-138 Warsaw, Poland; e.kopec@igichp.edu.pl

**Keywords:** pyridine, hydrazone, synthesis, antimicrobial activity, tuberculostatic activity, X-ray, DFT calculations

## Abstract

Four novel methyl 4-phenylpicolinoimidate derivatives of hydrazone have been synthesized and evaluated for their antimicrobial activity, including tuberculostatic activity. The compounds obtained are condensates of hydrazonamide or hydrazide with 5-nitro-2-furaldehyde or 5-nitro-2-thiophenecarboxaldehyde. The antimicrobial activity of the tested compounds varied. Compound **3b** exhibited significant activity against the tested Gram-positive bacteria (7.8–250 µg/mL). The results of structural tests revealed that the compound is the only one obtained in the form of a Z isomer. Tuberculostatic activity tests showed higher activity of derivatives **3a** and **4a** containing nitrofuran systems (MICs 3.1–12.5 µg/mL). This research allowed us to identify hydrazone **3b** as a starting point for further optimization in the search for antimicrobial drugs. Likewise, compound **4a** appears to be a good guiding structure for use in future research on new anti-tuberculosis drugs.

## 1. Introduction

An increase in the number of infections caused by resistant strains of pathogenic microorganisms has been observed for several decades. Tuberculosis is an infectious disease caused by *Mycobacterium tuberculosis* [1,2,3,4]. Its treatment requires the combined use of several chemotherapeutic agents and lasts up to 24 months [5,6,7]. Currently, in Europe, the total number of infections caused by tuberculosis bacilli remains unchanged, while the number of infections caused by multidrug-resistant strains (MDR-TB) is significantly rising. The treatment of tuberculosis is difficult because the most efficient drugs, such as isoniazid (INH) or rifampicin (RMP), lose their effectiveness against resistant strains. As a consequence, it becomes necessary to extend treatment and use second-line drugs with greater toxicity (e.g., ethionamide, cycloserine) [8,9]. According to the World Health Organization, 5.8 million people fell ill with tuberculosis in 2020. Half a million were cases resistant to RMP (one of the first-line drugs), 48% of which were also MDR-TB. These numbers may change dramatically as a result of increased migration across the continent Unfortunately, in 2021, only one in three patients entered treatment while 1.3 mln patients died [10].

The same is true of other infectious diseases caused by bacteria and fungi. Such infections remain a major health problem due to their resistance to currently used chemotherapeutic agents. Methicillin-resistant *Staphylococcus aureus* (MRSA) and vancomycin-resistant strains of *Enterococcus* (VRE) are particularly dangerous, causing the most lethal hospital infections. A disturbing fact is the growing resistance of Gram-negative bacteria to carbapenems and polymyxins, which are used in therapy as the last resort. The reasons for this phenomenon are attributed to the excessive exposure of the entire community to antibiotics and incorrect treatments of infectious diseases [11,12,13,14,15]. These phenomena reveal an urgent need for new antimicrobial drugs [10].

**Figure 1:**
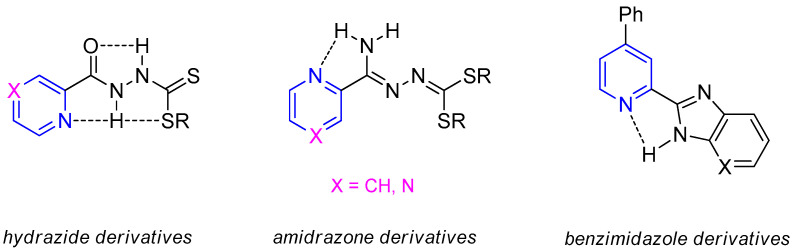
Structures of pyridine and pyrazine derivatives with proven activity against *M. tuberculosis*.

Many research studies have shown the biological potential of compounds with a pyridine or pyrazine rings, especially their anti-tuberculosis and anti-microbial activity [16,17,18,19,20,21]. Such systems include clinically used chemotherapeutic agents such as INH and pyrazinamide (PZA). Our previous studies showed that some hydrazinecarbodithioic acid esters and amides derived from pyridinamidrazone or pyrazinamidrazone and pyridincarbohydrazide or pyrazincarbohydrazide exhibit significant anti-tuberculosis activity [22,23,24,25]. The most active compounds from this group showed effectiveness against standard and resistant strains of *M. tuberculosis* with minimal inhibitory concentration (MIC) values in the range of 3.1–12.5 µg/mL (Figure 1). We also observed the same level of activity for benzimidazoles substituted at the C-2 position with a 4-phenylpicolin moiety [26,27,28]. We assumed that the flatness of these molecules may be a prerequisite for their activity [29,30].

**Figure 2:**
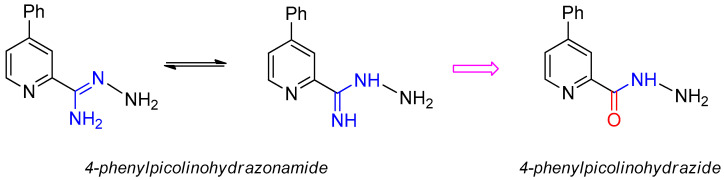
Structures of the obtained 4-phenylpicolinohydrazonamide and its isoster, 4-phenylpicolinohydrazide.

In the present study, we have obtained a series of hydrazone derivatives of 4-phenylpicolinohydrazonamide and 4-phenylpicolinohydrazide. The positive influence of the nitrofuryl moiety on antimicrobial activity, known from the literature and clinical practice [31,32], prompted us to use this structural moiety in the designed compounds. The obtained compounds are condensates of 4-phenylpicolinohydrazonamide or 4-phenyl-picolinohydrazide with 5-nitro-2-furaldehyde or 5-nitro-2-thiophenecarboxaldehyde and are two pairs of analogs differing in only one atom (oxygen or sulfur) in a five-membered heterocyclic ring. Because the classical isoster of the N-H group is the oxygen atom [33], we wanted to investigate whether and how the replacement of the NH moiety in hydrazonamides with oxygen in hydrazides would affect biological activity (Figure 2). In view of the wide spectrum of biological activities of hydrazide derivatives, including antimicrobial activity against Gram-positive and -negative bacteria [34], as well as tuberculostatic activity [35], we decided to obtain not only hydrazonamide derivatives but also hydrazide analogs.

## 2. Materials and Methods

### 2.1. Chemistry

All materials and solvents were of analytical reagent grade (Sigma-Aldrich-Merck KgaA, Darmstadt, Germany). Thin-layer chromatography was performed on Merck silica gel 60F_254_ plates and visualized with UV light. The results of elemental analyses (%C, H, N) for all of the obtained compounds were in agreement with the calculated values that were within the ±0.4 % range. The ^1^H and ^13^C NMR spectra in CDCl_3_ or DMSO-*d*_6_ were recorded on Varian Unity Plus (500 MHz) and Varian Gemini (200 MHz) instruments (Varian Medical Systems, Palo Alto, CA, USA). IR Spectra (KBr) were determined as KBr pellets of the solids on a Satellite FT-IR spectrophotometer (Bruker, Madison, WI, USA). Melting points were determined using a Stuart SMP30 apparatus (Stone, Staffordshire, UK) and were retained without any corrections.

#### 2.1.1. Methyl 4-Phenylpicolinimidate

To a solution of 4-phenylpicolinonitrile (1.8 g, 10 mmol) in methanol (30 mL) was added DBU (1,8-diazabicyclo[5.4.0]undec-7-ene, 1 mL, 7 mmol); the mixture was refluxed for 1 h. Then, the solvent was evaporated and the oily residue was treated with cyclohexane and SiO_2_ (1 g) and stirred for 5 h. The silica gel was filtered off and the solvent evaporated. The purified oily product crystallized after cooling, giving straw crystals (1.8 g, 85%): m.p. 80–82 °C; IR (KBr): 3298 (υ N-H), 3053, 2947 (υ C-H), 1654 (υ C=N), 1591 (δ N-H), 1440, 1358 (υ C=C), 1081 (υ C-O), 964 (δ C-H), 871, 758, 704 (γ C-H) cm^−1^; ^1^H NMR (200 MHz, DMSO-*d*_6_): δ 3.93 (s, 3H, CH_3_), 6.95 (dd, 1H, pyridine, J^1^ = 5 Hz, J^2^ = 2 Hz), 6.89–7.25 (m, 5H, PhH), 8.05 (s, 1H, pyridine), 8.36 (dd, 1H, pyridine, J^1^ = 5 Hz, J^2^ = 1 Hz), 10.04 (s, 1H, NH) ppm; Anal. Calcd. for C_13_H_12_N_2_O (212.09): C, 73.56; H, 5.70; N, 13.20; Found: C, 73.25; H, 5.37; N, 13.52. All data are consistent with a description in the literature [36].

#### 2.1.2. 4-Phenylpicolinohydrazonamide (**1**)

Methyl 4-phenylpicolinimidate (2.1 g, 10 mmol) was dissolved in methanol (10 mL), 98% hydrazine hydrate (3 mL, 95 mmol) was added, and the solution was refluxed for 1 h. Then, the mixture was cooled and water (30 mL) was added. The precipitate was filtered off and recrystallized using benzene, giving the compound **1** as a yellow solid (1.8 g, 85%): m.p. 110–111 °C; IR (KBr): 3444, 3395, 3351, 3310 (υ N-H), 3058, 2923 (υ C-H), 1646, 1597 (υ C=N), 1541 (δ N-H), 1467, 1427 (υ C=C), 883, 752, 696 (γ C-H) cm^−1^; ^1^H NMR (500 MHz, CDCl_3_): δ 4.65 (s, 2H, NH2 + D_2_O exchangeable), 5.36 (s, 2H, NH_2_ + D_2_O exchangeable), 7.44–7.55 (m, 4H, Ph), 7.71–7.77 (m, 2H, 1H Ph and 1H pyridine), 8.30 (s, 1H, pyridine), 8.58 (d, 1H, pyridine, J = 5 Hz) ppm; ^13^C NMR (175 MHz, CDCl_3_): δ 117.02, 120.06, 128.55 (2C), 129.46 (2C), 129.55, 138.00, 148.34, 148.90, 151.40, 157.85 ppm; Anal. Calcd. for C_12_H_12_N_4_ (212.11): C, 67.90; H, 5.70; N, 26.40; Found: C, 68.16; H, 5.41; N, 26.12.

#### 2.1.3. 4-Phenylpicolinohydrazide (**2**)

Methyl 4-phenylpicolinimidate (3.3 g, 17 mmol) was dissolved in methanol (15 mL) and 10% HCl (15 mL) was added while cooling. The solution turned clear at first and then became cloudy. After 0.5 h, the precipitate of methyl ester was filtered off and washed with saturated NaHCO_3_ solution and then with water. Then, ethanol (5 mL) was added to the precipitate and 98% hydrazine hydrate (3 mL, 95 mmol). The solution was refluxed for 1 h. The mixture was cooled and water (30 mL) was added. The precipitate of hydrazide was filtered off and recrystallized using ethanol, giving the title compound **2** as yellow solid (2.3 g, 85%): m.p. 106–107 °C; IR (KBr): 3412, 3315, 3267 (υ N-H), 3062 (υ C-H), 1685 (υ C=O), 1598, 1542 (δ N-H), 1510, 1498 (υ C=C), 962 (δ C-H), 764, 705, 690 (γ C-H) cm^−1^; ^1^H NMR (200 MHz, DMSO-*d_6_*): δ 4.62 (d, 2H, NH_2_ + D_2_O exchangeable, J = 4 Hz), 7.47–7.60 (m, 3H, Ph), 7.83–7.90 (m, 2H, 1H Ph and 1H pyridine), 8.24 (s, 1H, pyridine), 8.66 (d, 1H, pyridine, J = 5 Hz), 9.97 (br s, 1H, NH + D_2_O exchangeable) ppm; Anal. Calcd. for C_12_H_11_N_3_O (213.09): C, 67.59; H, 5.20; N, 19.71; Found: C, 67.70; H, 5.34; N, 19.40.

#### 2.1.4. General Procedure for the Synthesis of Hydrazones (**3a**,**b**–**4a**,**b**)

Compound **1** (0.21 g, 1 mmol) or compound **2** (0.21 g, 1 mmol) was dissolved in methanol (10 mL) and an appropriate aldehyde was added (1 mmol). The mixture was refluxed for 0.5 h and then left to crystallize. The precipitate was filtered off and recrystallized.

##### (*E*)-*N′*-((5-Nitrofuran-2-yl)methylene)-4-phenylpicolinohydrazonamide (**3a**)

Starting from 5-nitrofuran-2-carbaldehyde (0.14 g), compound **3a** was obtained as an orange solid (0.27 g, 80%): m.p. 207–208 °C (dioxane-water 1:1); IR (KBr): 3390, 3323 (υ N-H), 3163, 2923 (υ C-H), 1613 (υ C=N), 1578 (υ NO_2_), 1513, 1461 (υ C=C), 1351 (υ NO_2_), 1304, 1181 (υ C-O), 1008 (δ C-H), 810, 756 (γ C-H) cm^−1^; ^1^H NMR (200 MHz, DMSO-*d*_6_): δ 7.41–7.63 (m, 6H, 2H Ph and 1H CH and 1H furan and 2H NH_2_ + D_2_O exchangeable), 7.81–7.93 (m, 4H, 3H Ph and 1H furan), 8.44 (s, 1H, pyridine), 8.50 (m, 1H, pyridine), 8.75 (d, 1H, pyridine, J = 5 Hz) ppm; ^13^C NMR (175 MHz, DMSO-*d*_6_): δ 114.86, 115.64, 119.31, 123.87, 127.42 (2C), 129.85 (2C), 130.07, 137.31, 142.19, 148.60, 149.95, 150.87, 152.24, 154.14, 158.87 ppm; Anal. Calcd. for C_17_H_13_N_5_O_3_ (335.10): C, 60.89; H, 3.91; N, 20.89; Found: C, 60.93; H, 3.60; N, 20.73.

##### (*Z*)-*N′*-((5-Nitrothiophen-2-yl)methylene)-4-phenylpicolinohydrazonamide (**3b**)

Starting from 5-nitrothiophene-2-carbaldehyde (0.16 g), compound **3b** was obtained as a yellow solid (0.30 g, 85%): m.p. 156–157 °C (dioxane-water 1:1); IR (KBr): 3448, 3303 (υ N-H), 1618 (υ C=N), 1569 (υ NO_2_), 1524 (υ N-H), 1489, 1421 (υ C=C), 1328 (υ NO_2_), 1012, 984 (δ C-H), 815, 753, 686 (γ C-H) cm^−1^; ^1^H NMR (200 MHz, DMSO-*d*_6_): δ 7.56–7.67 (m, 4H, 3H Ph and 1H thiophene), 7.75 (s, 1H, NH + D_2_O exchangeable), 7.83 (s, 1H, NH + D_2_O exchangeable), 8.00–8.14 (m, 3H, 2H Ph and 1H CH), 8.17 (d, 1H, thiophene, J = 4 Hz), 8.36 (s, 1H, pyridine), 8.80 (d, 1H, pyridine, J = 5 Hz), 8.90–8.91 (m, 1H, pyridine) ppm; ^13^C NMR (175 MHz, DMSO-*d*_6_): δ 114.89, 115.57, 119.31, 123.85, 127.40 (2C), 129.83 (2C), 130.05, 137.33, 142.23, 148.61, 149.93, 150.88, 152.25, 154.09, 158.83 ppm; Anal. Calcd. for C_17_H_13_N_5_O_2_S (351.08): C, 58.11; H, 3.73; N, 19.93; Found: C, 58.37; H, 3.94; N, 20.17.

##### (*E*)-*N′*-((5-Nitrofuran-2-yl)methylene)-4-phenylpicolinohydrazide (**4a**)

Starting from 5-nitrofuran-2-carbaldehyde (0.14 g), compound **4a** was obtained as an orange solid (0.29 g, 88%): m.p. 285–287 °C (methanol); IR (KBr): 3277 (υ N-H), 3106 (υ C-H), 1689 (υ C=O), 1533 (υ NO_2_), 1468 (υ C=C), 1354 (υ NO_2_), 1248, 1137 (υ C-O), 966 (δ C-H), 810, 766 (γ C-H) cm^−1^; ^1^H NMR (500 MHz, DMSO-*d*_6_): δ 7.28 (d, 1H, furan, J = 4 Hz), 7.51–7.58 (m, 3H Ph), 7.81 (d, 1H, furan, J = 4 Hz), 7.90 (d, 1H, Ph, J = 7 Hz), 8.03 (d, 1H, furan, J = 4 Hz), 8.38 (s, 1H, pyridine), 8.64 (s, 1H, CH), 8.79 (d, 1H, pyridine, J = 5 Hz, 12.78 (1H, NH with D_2_O exchangeable) ppm; ^13^C NMR (175 MHz, DMSO-*d*_6_): δ 115.07, 115.55 (2C), 120.54, 124.99, 127.53 (2C), 129.86 (2C), 130.27, 136.88, 137.43, 149.12, 149.83, 150.40, 152.36, 161.33 ppm; Anal. Calcd. for C_17_H_12_N_4_O_4_ (336.09): C, 60.71; H, 3.60; N, 16.66; Found: C, 60.68; H, 3.85; N, 16.99.

##### (*E*)-*N′*-((5-Nitrothiophen-2-yl)methylene)-4-phenylpicolinohydrazide (**4b**)

Starting from 5-nitrofuran-2-carbaldehyde (0.16 g), compound **4b** was obtained as an orange solid (0.30 g, 85%): m.p. 220–222 °C (methanol); IR (KBr): 3249 (υ N-H), 3108, (υ C-H), 1686 (υ C=O), 1539 (υ NO_2_), 1522, 1435 (υ C=C), 1335 (υ NO_2_), 1047, 1029 (δ C-H), 761, 644 (γ C-H) cm^−1^; ^1^H NMR (500 MHz, DMSO-*d*_6_): δ 7.54–7.56 (m, 3H Ph), 7.75–7.76 (m, 2H Ph), 8.87–8.90 (m, 1H, thiophene), 8.53 (s, 1H, CH), 8.62–8.65 (m, 2H, 1H pyridine and 1H thiophene), 8.69–8.71 (m, 1H pyridine), 8.94–8.96 (m, 1H, pyridine), 11.78 (1H, NH + D_2_O exchangeable) ppm; ^13^C NMR (175 MHz, DMSO-*d*_6_): δ 120.50, 125.00, 127.54 (2C), 129.89 (2C), 130.24, 130.31, 130.98, 136.84, 142.83, 147.14, 149.38, 149.84, 150.38, 151.43, 161.20 ppm; Anal. Calcd. for C_17_H_12_N_4_O_3_S (352.06): C, 57.95; H, 3.43; N, 15.90; Found: C, 57.56; H, 3.33; N, 16.12.

### 2.2. ADMET

The compounds were analyzed for their pharmacokinetic properties, drug-likeness and absorption. An ADME (Absorption, Distribution, Metabolism, and Excretion) analysis was performed using the SwissADME service (Swiss Institute of Bioinformatics 2021): a free web tool to evaluate pharmacokinetics, drug-likeness and medicinal chemistry friendliness of small molecules, and BOILED-Egg, to predict the gastrointestinal absorption and brain penetration of the molecules [37]. The bioavailability radar for each compound was determined. ProTOX II provides predictions of the toxicities of compounds [38].

### 2.3. Biological Activities

#### 2.3.1. In Vitro Antimicrobial Activity Assay

The antibacterial and antifungal activities of the tested compounds were screened by microdilution broth method using Mueller-Hinton broth and Mueller-Hinton broth with 2% glucose for growth of bacteria and fungi, respectively. The minimal inhibitory concentrations (MICs) of the tested derivatives were evaluated for the panel of the reference microorganisms, including Gram-negative bacteria (*Proteus mirabilis* ATCC 12453, *Escherichia coli* ATCC 25922, and *Klebsiella pneumoniae* ATCC 13883, Gram-positive bacteria (*Micrococcus luteus* ATCC 10240, *Staphylococcus aureus* ATCC 25923, *Staphylococcus epidermidis* ATCC 12228, *Bacillus subtilis* ATCC 6633, and *Bacillus cereus* ATCC 10876) and fungi (*Candida parapsilosis* ATCC 22019) (LGC Standards, Teddington, Middlesex, UK). The antimicrobial assays were performed in the same manner as in our previous research [39]. Vancomycin, Ciprofloxacin, and fluconazole were used as standard drugs. Each experiment was performed in triplicate. Representative data are presented.

#### 2.3.2. Tuberculostatic Activity Assay

The newly synthesized hydrazones were tested in vitro for their tuberculostatic activity toward the *M. tuberculosis* standard strain, H_37_Rv (National Tuberculosis and Lung Diseases Research Institute, Warsaw, Poland) and two native strains isolated from tuberculosis patients (National Tuberculosis and Lung Diseases Research Institute, Warsaw, Poland): Spec. 210, resistant to p-aminosalicylic acid (PAS), INH, ethambutol (ETB); and RMP and Spec. 192, fully sensitive to the administrated tuberculostatic drugs. Investigations were performed by a classical test-tube method described in detail earlier [39]. Each experiment was performed in triplicate. Representative data are presented.

### 2.4. X-ray Study

Monocrystal X-ray diffraction measurements of all compounds were made using a Bruker SMART APEXII CCD Diffractometer (Bruker AXS Inc., Madison, WI, USA) with CuKα radiation at 100 K. The diffraction data were processed with SAINT ver. 8.34 A, SADABS ver. 2014/4 and XPREP ver. 2014/2 (Bruker AXS Inc., Madison, WI, USA). The structures were determined with the ShelXT 2018/2 solution program (Version 2018/2, 2018, Göttingen, Germany) [40] and refined with ShelXL 2018/3 [41]. For visualization, ShelXle [42] was used. The addition and refinement of hydrogen atoms has been described in the previous work [43].

CCDC 2154198, 2154201, 2154207 and 2154204 contain the supplementary crystallographic data for this paper. The data are provided free of charge by The Cambridge Crystallographic Data Center via www.ccdc.cam.ac.uk/structures (accessed on 23 February 2022).

### 2.5. DFT Calculations

Quantum calculations were performed with GAMESS-US software [44] using DFT/B3LYP [45,46,47] with the base functions 6–311 G (d,p) to optimize the geometry of the studied compounds, taking into account the solvent effect of water, using the polarizable continuum model.

## 3. Results and Discussion

### 3.1. Synthesis

**Figure 3:**
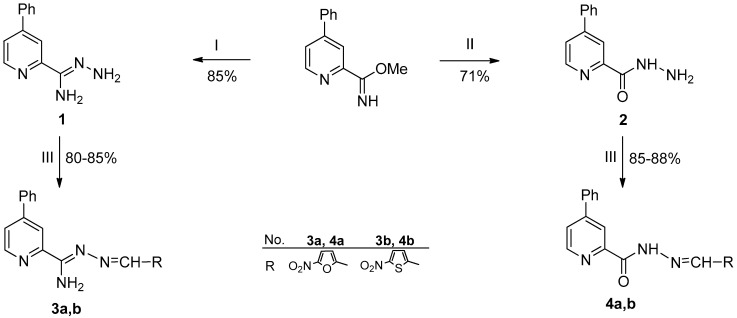
Synthesis of 4-phenylpicolinonitrile derivatives **1**–**4a**,**b**. Reagents and conditions: (I) 98% NH_2_NH_2_·H_2_O, MeOH, reflux; (II) stage (1) MeOH, 10% HCl, ice bath, NaHCO_3_, CH_2_Cl_2_, MgSO_4_; stage (2) 98% NH_2_NH_2_·H_2_O, EtOH reflux; (III) RCHO, MeOH, reflux.

The initial compound 4-phenyl-2-cyanopyridine, refluxed for 1 h with DBU in methanol, gave methyl 4-phenylpicolinimidate with 85% yield (Figure 3). Amidrazone **1** was obtained by refluxing methyliminoester with hydrazine hydrate in methanol for 1 h (yield 85%). Treatment of the iminoester with 10% HCl in methanol yielded a methyl ester which, upon reaction with hydrazine hydrate in ethanol, gave hydrazide **2** (71%). Hydrazonamide **1**, when refluxed for 0.5 h in methanol with appropriate aldehydes (2-nitrofuryl, 2-nitrothiophene), reacted to condensates **3a**,**b** (yields 80% and 85% respectively) as well as hydrazide **2** to hydrazones **4a**,**b** (yields 88% and 85%, respectively). All the newly synthesized compounds were characterized by IR, ^1^H NMR and ^13^C NMR spectra and elemental analysis. The results from the spectral analysis were in accordance with the assigned structures.

### 3.2. ADMET Analysis

**Figure 4:**
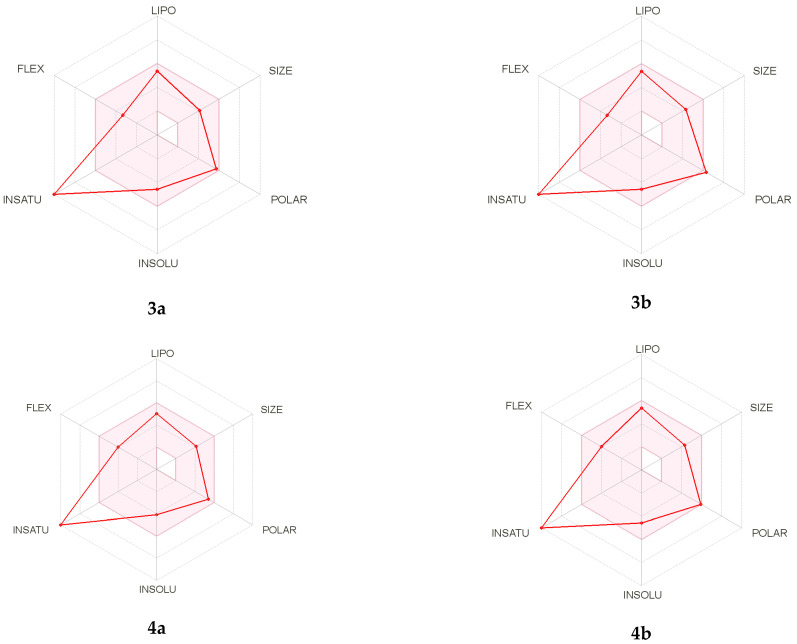
Bioavailability radars for **3a**, **3b**, **4a** and **4b**.

**Figure 5:**
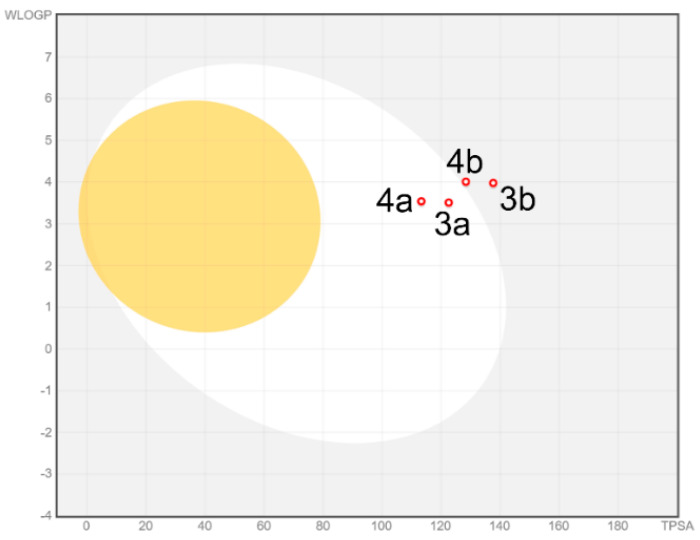
BOILED-Egg diagram for all compounds (lipophilicity (WLOGP) and polarity (tPSA), human intestinal absorption, white area; and blood–brain barrier permeation, yellow area.

Bioavailability radars were performed for all studied compounds (Figure 4). The ranges for the plot are: lipophility (LIPO) within the range −0.7 < XlogP3 < +5.0; molecular weight (SIZE) is 150 g/mol < MW < 500 g/mol; polarity (POLAR) is 20 Å^2^ < TPSA < 130 Å^2^; insolubility (INSOLU) is 0 < logS < 6;—insaturation (INSATU) is 0.25 < fraction Csp3 < 1; and flexibility (FLEX) are 0 < num. rotatable bonds < 9. For drug-like properties, the compounds were found to have a good bioavailability score (0.55). All compounds complied with the rules of Lipinski [48], Ghose [49], Egan [50], Veberm [51] and Muegge [52]. This means they are good drug candidates. In the BOILED-Egg diagram (Figure 5), compounds **3a** and **4a** showed absorption via the gastrointestinal tract, which would make them effective drugs. They are not substrates of P-gp, which means they are good candidates against multidrug resistant cancer cells. The analysis showed that all tested compounds could be tested, while in terms of absorption in the gastrointestinal tract, two of them were very interesting (**3a** and **4a**).

Service ProTox II classified **4a** at toxicity class 4 (predicted LD_50_: 1500 mg/kg, i.e., harmful if swallowed (300 < LD_50_ ≤ 2000)), **4b** at toxicity class 5 (predicted LD_50_: 3506 mg/kg, i.e., may be harmful if swallowed (2000 < LD_50_ ≤ 5000)), **3a** at toxicity class 4 (predicted LD_50_: 750 mg/kg) and **3b** at toxicity class 3 (predicted LD_50_: 200 mg/kg, i.e., toxic if swallowed (50 < LD_50_ ≤ 300)) [38].

### 3.3. Antimicrobial Activity

All of the obtained compounds were evaluated for their in vitro antimicrobial activity. The first screening of antimicrobial activity was performed on representatives of Gram-positive bacteria, i.e., *Staphylococcus epidermis*, *S. aureus*, *Micrococcus luteus*, *Bacillus subtilis*, *B. cereus*; Gram-negative bacteria, i.e., *Escherichia coli* and *Klebsiella pneumonia*; and fungi, i.e., *Candida parapsilosis*. Ciprofloxacin, vancomycin, and fluconazole were used as reference drugs (Table 1).

The results given in Table 1 indicate that the inhibitions by compounds were lower than those of standard drugs. The most promising compound turned out to be **3b**, which demonstrated very strong anti-staphylococcal activity (MIC < 10 µg/mL), good activity against spore-forming bacilli (MIC in the range 31.3–125 µg/mL), and moderate anti-micrococci bioactivity. In general, compounds **3b** and **4b** with sulfur-containing moieties showed higher activity against the tested Gram-positive bacteria in comparison to compounds **3a** and **4a** with 5-nitro-furan-2-yl moiety (except with *S. aureus*, against which they showed mild activity). The possible reason for the antibacterial action of these compounds may be that they bind to the membranes of microorganisms through hydrogen bonding with sulfur, increasing the time to complete cell division; thus, the generation time of the bacteria was prolonged. Moreover, **3a** and **3b** presented very mild bioactivity against Gram-negative bacteria, whereas **4a** and **4b** did not demonstrate bioactivity according to accepted definitions [53]. Thus, the antibacterial efficiency of the tested derivatives decreased in the following order: **3b** > **3a** > **4b** > **4a**. Moderate bioactivity was found against the representative yeast strain, irrespective of the kind of derivatives. The differences in biological activity of compounds to Gram-positive and -negative bacteria could be explained by the differences in their cell wall structure, and thus, in permeability. Peptydoglycan is major component (90%) of the Gram-positive cell wall. The presence in Gram-negative bacteria of an outer lipid biliayer containing lipopolysaccharide, porins, adhesions creates an additional barrier which must be overcome.

### 3.4. Tuberculostatic Activity

The newly obtained hydrazones were also tested for tuberculostatic activity against three strains of *M. tuberculosis*, i.e., the standard strain H37Rv and two native strains from patients, namely, Spec 210, which is resistant to clinically used anti-tuberculosis drugs (PAS, INH, ETB, RMP) and Spec 192, which is completely sensitive. INH and PZA were used as reference drugs. The tested derivatives showed varied tuberculostatic activity with MIC values in the range of 3.1–25 µg/mL (Table 2). However, some of them showed activity that was better than that of reference drug pyrazinamide, with activity against the standard H37Rv strain and susceptible strain Spec. 192 at a MIC level of 25 µg/mL, and against the resistance strain Spec. 210 at over 400 µg/mL, but also INH (MICs of 12.5 µg/mL and 25 µg/mL, respectively). Compound **3a** and **4a** with 5-nitro-furan-2-yl moieties showed good activity, with MIC values in a range of 3.1–12.5 µg/mL. Compound **3a**, i.e., an amidrazone derivative with a 5-nitro-furan-2-yl moiety, showed good activity with an MIC value of 6.2 µg/mL. Its activity was, respectively, four times higher against the standard and resistant strains than its sulfur-containing analog **3b** and two times higher against the sensitive strain compared to reference INH. This compound was four times more potent against resistant Spec. 210 than INH. Compound **4a**, a hydrazide derivative, showed even better activity with an MIC value of 3.1 µg/mL against both sensitive and resistant strains; it was eight times more active than its sulfur analog **4b** and four times more active than reference INH. It is noteworthy that for most compounds, the MIC values against the resistant strain were at the same level as those against the standard strain. One consequence of acquiring resistance is the necessity to use increased doses of chemotherapeutic agents. In our study, the resistant mycobacteria showed exactly the same sensitivity to the compounds tested by us as the non-resistant mycobacteria. This may mean that the tested compounds bypassed the molecular targets which are characteristic of classic anti-tuberculosis chemotherapeutic agents against which resistance has been demonstrated with strain Spec. 210 [54,55].

### 3.5. X-ray Study

**Figure 6:**
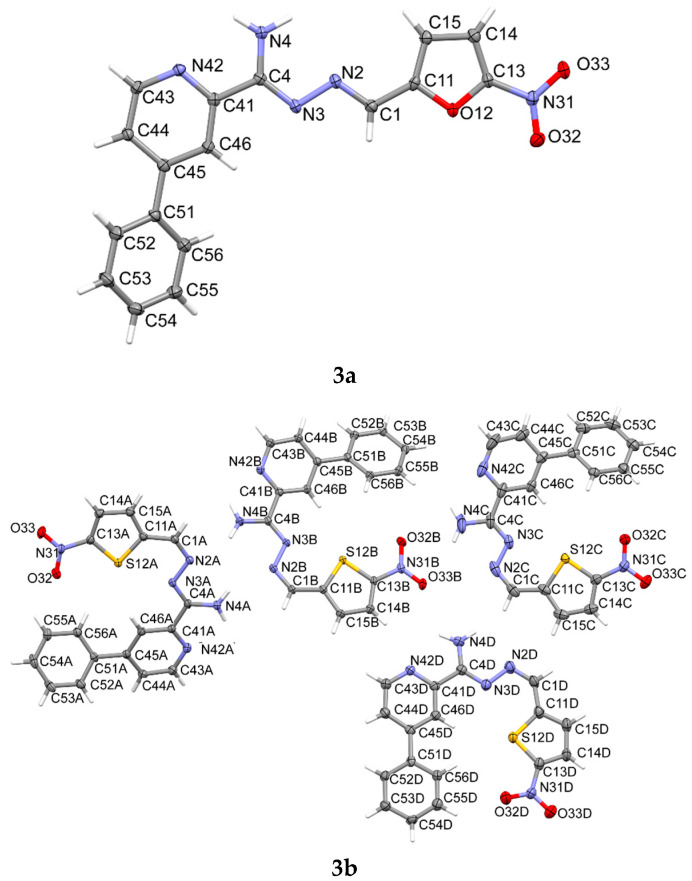

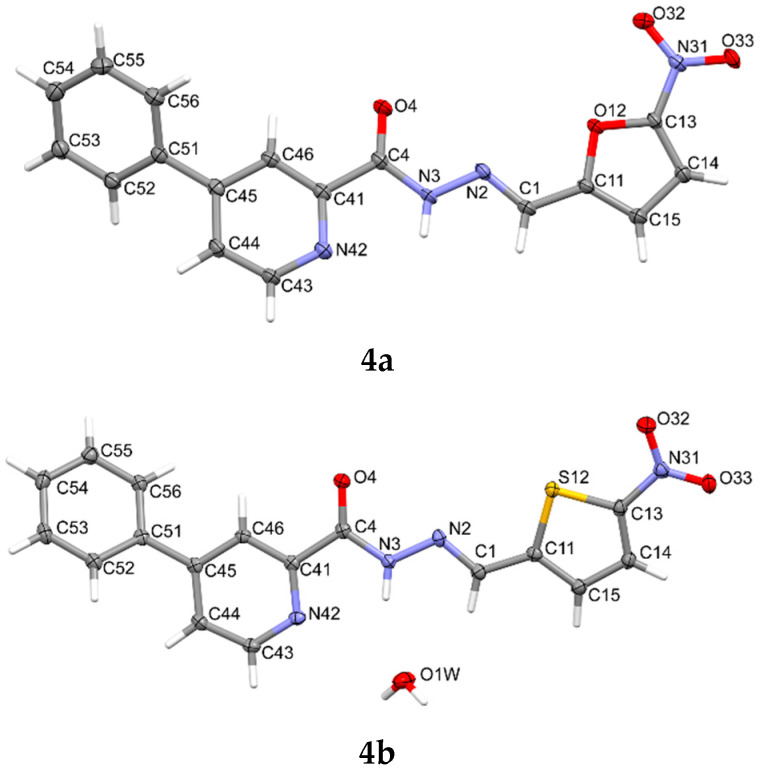
The molecular structures of compounds **3a**, **3b**, **4a** and **4b**, showing the atom-labeling schemes. Displacement ellipsoids are drawn at the 50% probability level, and H atoms are shown as small spheres of arbitrary radii. Drawings were prepared using the Mercury software.

**Figure 7:**
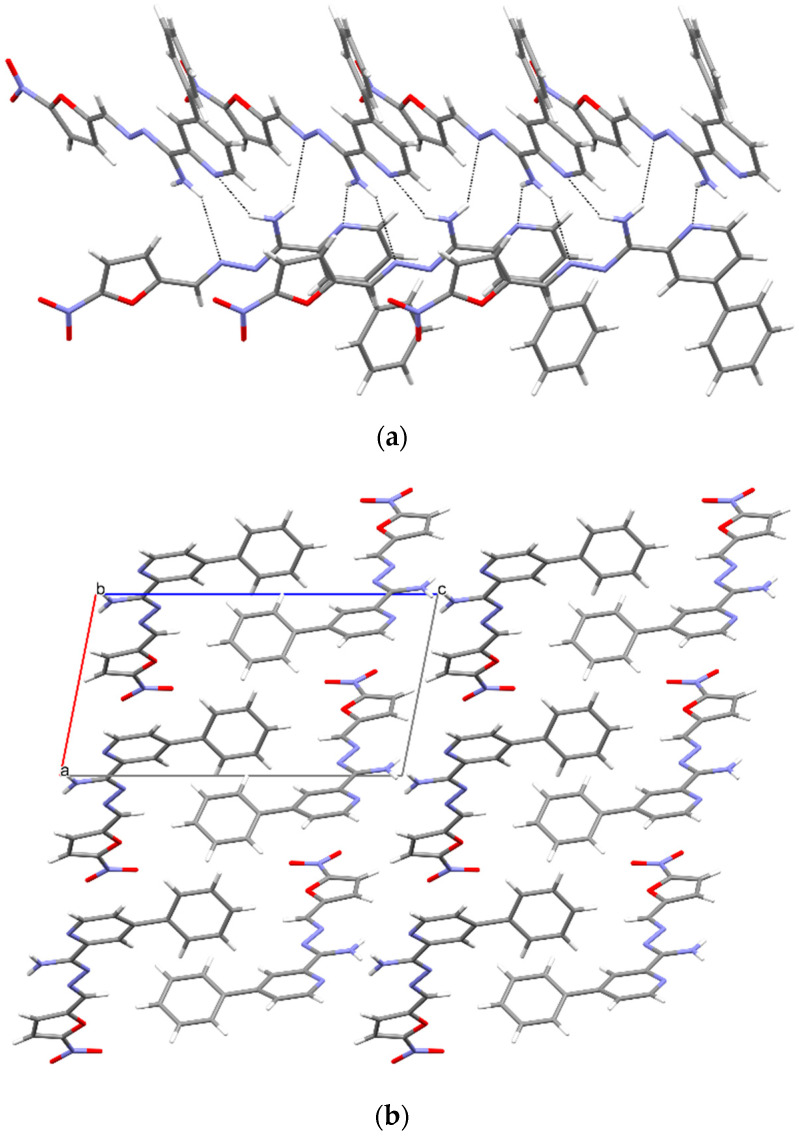
(**a**) Intermolecular hydrogen bonds in compound **3a**. (**b**) The crystal packing of compound **3a** (a, b, c—unit cell).

**Figure 8:**
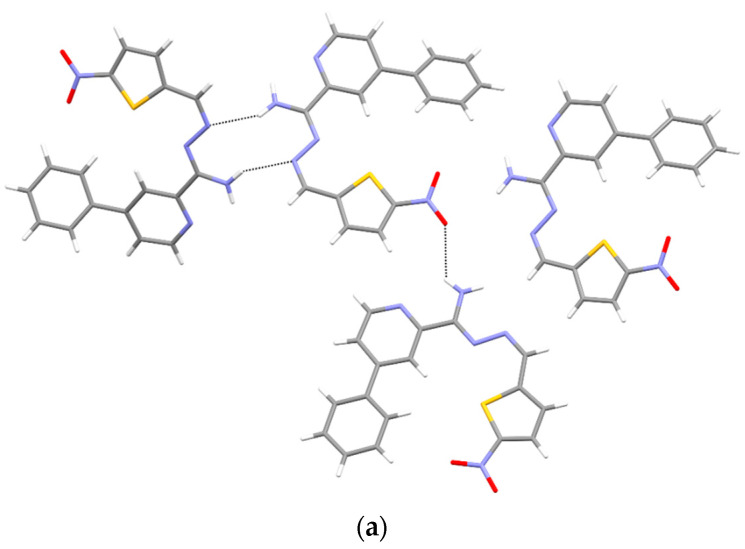

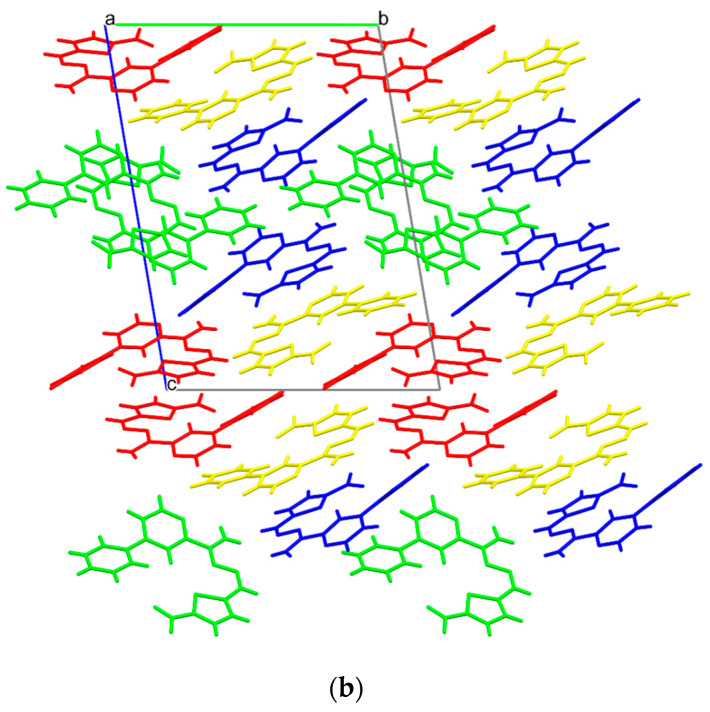
(**a**) Intermolecular hydrogen bonds in **3b**. (**b**) The packing of molecules in **3b** (a, b, c—unit cell).

**Figure 9:**
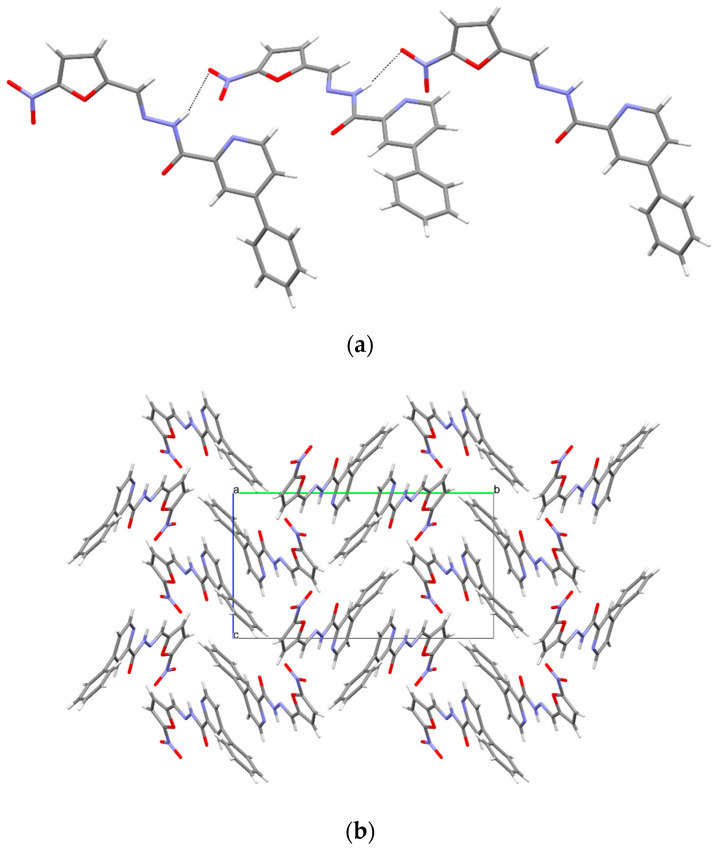
(**a**) Intermolecular hydrogen bonds in **4a**. (**b**) The packing of molecules in **4a** (a, b, c—unit cell).

**Figure 10:**
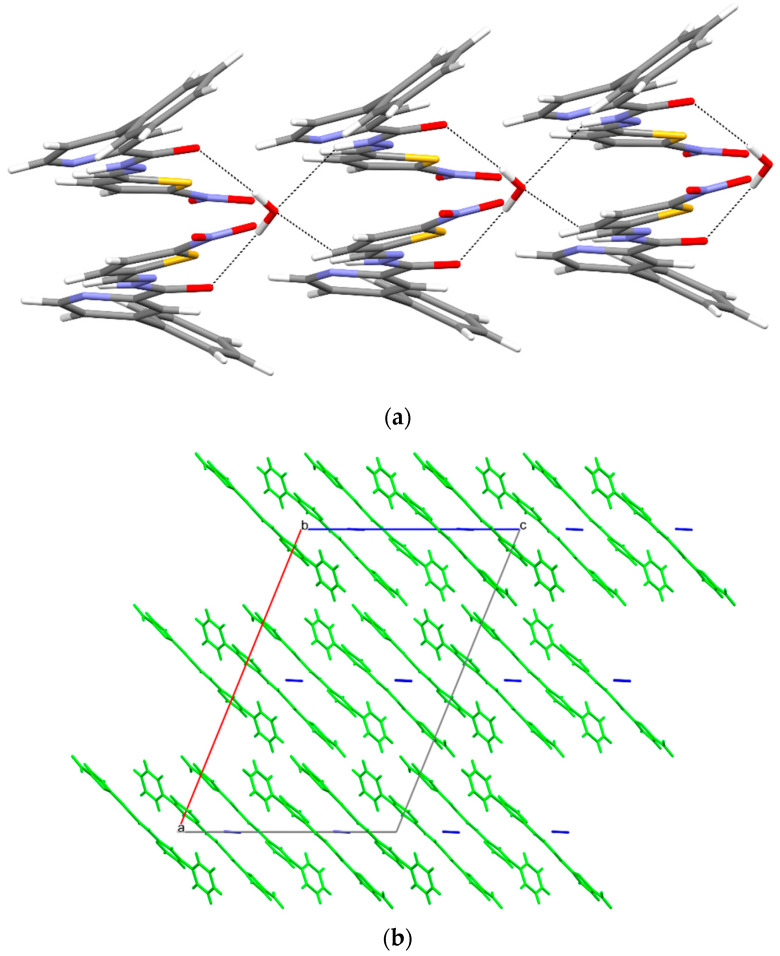
(**a**) Intermolecular hydrogen bonds in **4b**. (**b**) The packing molecules in **4b** (a, b, c—unit cell).

The significant differences in the activities of the studied compounds prompted us to look closely at their spatial structure by X-ray diffraction (Figure 6). Appropriate crystals were obtained by slow evaporation of a DMF-methanol mixture. The basic crystallographic data are presented in Table 3, while Figure 7, Figure 8, Figure 9 and Figure 10 show details of their crystal structures and packing. Powder diffraction diagrams confirmed that the crystals chosen for the structure analysis were representative of the entire powder sample (Figure S1). Crystals of compound **3b** revealed some twinning, which was difficult to resolve during data processing, and consequently, the data were reduced, as for a single crystal. Probably because of this unresolved twinning, the residual electron density was much higher for this structure (Table 3). Compounds **3a** and **3b** are hydrazonamide derivatives. In the case of compound **3a**, there was only one molecule in the asymmetric unit, as opposed to compound **3b**, in which there were four molecules. Moreover, compound **3b** was the only one with the Z conformation. In the case of hydrazide derivatives **4**, there was one molecule in an asymmetric unit. Additionally, a water molecule was built into the structure of compound **4b**.

In **3a**, the molecules form chains by means of N-H · N intermolecular hydrogen bonds (C2,2 (8) type, Figure 7, Table 4), resulting in a layered arrangement of the molecules in the crystals (Figure 7).

In structure **3b**, three of the four independent molecules are stabilized by intermolecular hydrogen N-H⋯N bonds (R2,2 (10)) and N-H⋯O ones (Figure 8, Table 5). The fourth molecule (denoted as C) serves as a space filler, as seen from higher displacement parameters of its atoms and standard uncertainties of respective bonds lengths and angles (Tables S1 and S2).

In structure **4a**, chains of C1,1 (9) hydrogen bonds are formed. In addition, the flat build of the molecules is stabilized by an intramolecular hydrogen bond of type N3-H⋯N42 (Figure 9, Table 6). The packing of the molecules resembles that most often found in aromatic (planar) systems.

In structure **4b**, rings of hydrogen bound molecules are formed [R3,4 (12)] and stabilized by water molecules, which, in turn, form chains running in direction [010] (Table 7, Figure 10). In addition, the flatness of the molecules is stabilized by N3-H⋯N42 hydrogen bonds. As a result, a layered structure stabilized by hydrogen bonds with water molecules is formed. In general, the packing resembles, to some degree, that observed in **4a** (Figure 10), despite the presence of water in **4b** and the different symmetries (space groups) in the two structures **4** (Table 3).

The molecular parameters of the described structures (Tables S1 and S2) are in good agreement with the values found for similar compounds (Scheme S1) in the crystal state (Cambridge Structural Database [56]). They confirm the conjugation system comprising C1-N2-N3-C4-C41(2-pyridine) chains, resulting in the shortening of formally singular N-N bonds to 1.37–1.40 Å (the longest values of about 1.50 Å were observed for singular N-N bonds—see Scheme S1) and the lengthening of formally double bonds (C1=N2 in 3 to 1.28–1.29 Å and N3=C4 in 4 to 1.31 Å). The coplanarity of the central chain fragment with the pyridine ring is secured both by conjugations, as evidenced by the shortening of C4-C41 bonds to about 1.50 Å (from 1.53 for pure aliphatic C4 substituents) and by intramolecular hydrogen bonds (N4-H⋯N42 in **3a**,**b** and N3-H⋯N42 in **4a**,**b**).

**Figure 11:**
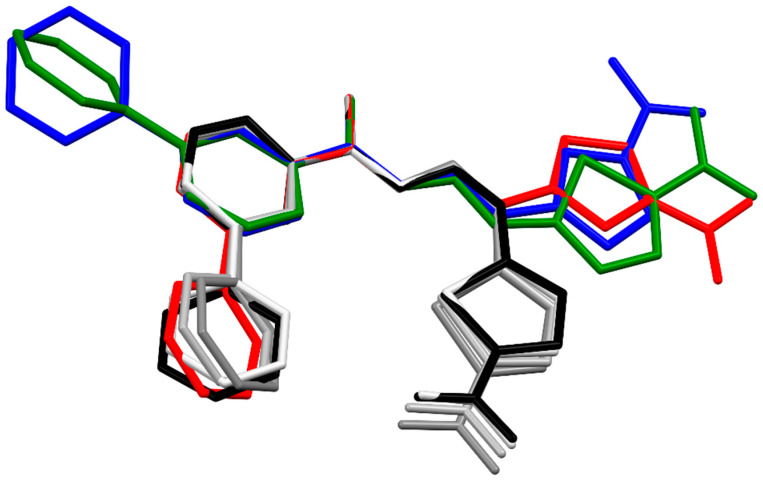
Overlay of molecules for all determined structures; **3a**—red, **3b**—light grey, grey, dark grey, black green and green, **4a**—blue, and **4b**—green. H atoms were omitted for clarity.

The superposition of all the molecules studied here shows general similarities in the conformations of their main chains (C1-N2-N3-C4-C41) (Figure 11). The different positions of the phenyl rings in structures **3** and **4** results from different orientations of the pyridine rings, which, in turn, is the result of different possible intramolecular hydrogen bonds, being N3-H⋯N(pyridine) in **3a**,**b** or N4-H⋯N(pyridine) in **4a**,**b**. Meanwhile, a wide range of phenyl ring rotations (Figure 9) reflects the significant freedom of their movements. On the other hand, the unique position of the nitrothiophene substituent in **3b** (Figure 8) results from its Z configuration in the C1=N2 bond, as compared to all other studied structures, which comprised E isomers (Figure 6). The consequence of the Z configuration in **3b** is the coiled shape of the molecules (best seen in Figure 8) instead of the elongated form observed for E isomers (Figure 6). We believe that flat molecules are easy to pack and therefore, in most cases, have one independent molecule in an asymmetric unit, while irregularly shaped (or even flat) molecules (like **3b**, in contrast to **3a** and **4a**,**b**) often need to form clusters of several molecules to pack efficiently. In the case of **3b**, there are four independent molecules in the crystal structure (Figure 8).

The most important difference between the studied compounds is the substituent at the C4 atom, being either an amine group (in **3a**,**b**) or an oxygen atom (in **4a**,**b**). The two substituents play the opposite role in the formation of strong hydrogen bonds (as a donor in **3** and an acceptor in **4**). This means that the two types of compounds should not interact with the same receptor (or at least not in the same way), as suggested by their similar tuberculostatic activity (Table 2). Still, at a glance, it was surprising that despite this difference, the central parts of both hydrazonamides (**3a**,**b**) and hydrazides (**4a**,**b**) stretched from C1 to the pyridine ring were approximately planar and had very similar shapes (Figure 11). The planarity is due to the conjugations (Table S1) aided by intramolecular hydrogen bonds N4-H⋯N(pyridine) in **3a**,**b** or N3-H⋯N(pyridine) in **4a**,**b**.

A detailed analysis of the crystal structures showed the conservative flat build of the central parts of all four studied compounds, despite the presence of different substituents at the C4 atom having the opposite functionality in hydrogen bonding (amine in **3a**,**b** and oxygen atom in **4a**,**b**). This difference concerns both the different hydrogen bonds of the two groups and the reversed positions of the pyridine ring, correlated with phenyl occupying another space. Still, due to conjugations and intramolecular hydrogen bonds, i.e., N4-H⋯N(pyridine) in **3a**,**b** and N3-H⋯N(pyridine) in **4a**,**b**, a rigid geometry of the central part of studied molecules was observed. One effect of the intramolecular hydrogen bonds in the case of **4** is the lack of another acidic H atom in the molecule which would be capable of interacting with a hypothetical receptor through a hydrogen bond. In addition, the hydrogen bond shields the carbonyl O atom from possible interactions as a hydrogen bond acceptor. However, in the case of **3a**,**b**, one acidic H atom remains free for intermolecular interactions as a hydrogen bond donor, which should be kept in mind when comparing the differences in the activity profiles of hydrazonamides (**3**) and analogous hydrazides (**4**).

Another interesting observation was that structures **4a** and its S-analog **4b** (with thiophene instead of the furan ring) were not isostructural. However, a search of the Cambridge Structural Database [56] showed that among 170 crystal structures comprising one thiophene ring in a molecule (see Supplementary Materials for the fragments tested and details of the procedure), 35 examples (i.e., about 20%) of exact furan analogs were detected, of which almost half (16) were isostructural, i.e., they had the same space group, very similar unit cells dimensions, molecular conformation and packing. Therefore, the dissimilarity of the crystal structures of **4a** and **4b** did not seem unusual, especially given that both the S (in thiophene ring) and O (in furan ring) did not show any important interactions or close contact in the studied structures. Of course, in the case of compounds **3a** and **3b**, they were only formally S, O analogs. In fact, they are geometrical isomers and cannot form similar crystal structures.

### 3.6. Ab-Initio

Interestingly, **3a** and **3b** (differing only in the heteroatom in the five-membered ring) assumed completely different configurations (Figure 11) in the crystalline state. This observation prompted us to perform energy calculations for both compounds in unfolded (E isomer) and bent (Z isomer) forms. We optimized the geometry of the isolated molecules, taking into account the solvent (water) effect using the polarizable continuum model (PCM). The geometries of the optimized **3a** (E) and **3b** (Z) molecules differed very little from the original crystallographic conformations. It turned out that in both cases, the isomers found in the crystal states represented lower energy. For **3a**, the energy of the unfolded (E) conformation was about 1.4 kcal/mol lower than that of the bent (Z) conformation. For **3b**, the relationship was the opposite: the bent conformation had a lower energy, i.e., about 4.6 kcal/mol. The energy differences were not large, especially in the first case, but they suggest that the final step of reaction III (Figure 3) is under thermodynamic control.

## 4. Conclusions

In conclusion, four novel hydrazone derivatives of 4-phenylpyridine were successfully synthesized from methyl 4-phenylpicolinoimidate. Tests of antimicrobial activity against Gram-positive bacteria, Gram-negative bacteria and yeast showed that compound **3b**, containing a nitrothiophene system in its structure and adopting a Z configuration at the N1=N2 bond, had the best activity against Gram-positive bacteria. Further studies are needed to evaluate the assumption that the promising profile of activity of **3b** is related to its Z configuration at the N1=C2 bond. Additionally, significant potential activity of hydrazone derivative **4a** with a nitrofuran system in its structure, assessed in vitro against *M. tuberculosis,* was detected.

The most important finding is that due to their significant potential action against the tested microorganisms, compounds **3b** and **4a** may be good starting structures for further research on the development of new antimicrobial drugs.

## Figures and Tables

**Table 1:** In vitro antimicrobial activity of compounds ^1,2^.

Compound	3a	3b	4a	4b	CIP ^2^	VAN ^2^	FCZ ^2^
Microorganism	MIC ^1^ [µg/mL]						
	Gram-positive bacteria						
*S. aureus* ATCC 25923	1000	7.8	1000	1000	0.49	0.98	-
*S. epidermidis* ATCC 12228	0.49	7.8	3.9	0.98	0.49	0.98	-
*M. luteus* ATCC 10240	500	250	1000	62.5	0.98	0.12	-
*B. subtilis* ATCC 6633	0.49	31.3	125	31.3	0.03	0.24	-
*B. cereus* ATCC 10876	250	125	125	1000	0.12	0.98	-
	Gram-negative bacteria						
*E. coli* ATCC 25922	1000	1000	>1000	>1000	0.004	-	-
*K. pneumoniae* ATCC 13883	1000	>1000	>1000	>1000	0.12	-	-
*P. mirabilis* ATCC 12453	>1000	1000	>1000	>1000	0.03	-	-
	Yeast						
*C. parapsilosis* ATCC 22019	250	250	250	250	-	-	1.95

^1^ Each experiment was performed in triplicate. Representative data are presented. ^2^ CIP ciprofloxacin, VAN vancomycin, FCZ fluconazole.

**Table 2:** Tuberculostatic activity of tested compounds ^1,2,3^.

Compd.	MIC ^1^ [µg/mL]		
	H_37_Rv ^2^	Spec. 192	Spec. 210
**3a**	**6.2**	**6.2**	**6.2**
**3b**	25	25	25
**4a**	**3.1**	**12.5**	**3.1**
**4b**	25	25	25
INH ^3^	12.5	12.5	25
PZA ^3^	25	25	>400

The values obtained for the most potent compounds are marked in bold. ^1^ Minimal inhibiting concentrations were determined by a classical test-tube method of successive dilution. Each experiment was performed in triplicate. Representative data are presented. ^2^ *M. tuberculosis* H_37_Rv, Spec. 192, Spec. 210. ^3^ INH isoniazid; PZA pyrazinamide.

**Table 3:** Crystal data, data collection and refinement details.

	**3a**	**3b**	**4a**	**4b**
Chemical formula	C_17_H_13_N_5_O_3_	C_17_H_13_N_5_O_2_S	C_17_H_12_N_4_O_4_	2(C_17_H_12_N_4_O_3_S)·H_2_O
*M*_r_	335.32	351.38	336.31	722.75
Crystal system, space group	Monoclinic, *P*2_1_	Triclinic, *P*-1	Monoclinic, *P*2_1_/*c*	Monoclinic, *C*2/*c*
*a*, *b*, *c* (Å)	7.9576 (1), 6.8200 (1), 14.7329 (3)	9.3981 (2), 16.2901 (4), 21.5857 (6)	9.8950 (4), 16.6918 (7), 10.2708 (5)	27.8383 (15), 6.6147 (4), 18.6149 (10)
α, β, γ (°)	90, 101.1060 (4), 90	78.623 (1), 81.641 (1), 75.018 (1)	90, 114.976 (1), 90	90, 112.197 (1), 90
*V* (Å^3^)	784.59 (2)	3114.04 (13)	1537.74 (12)	3173.8 (3)
*Z*	2	8	4	4
Crystal size (mm)	0.34 × 0.18 × 0.17	0.57 × 0.24 × 0.10	0.23 × 0.18 × 0.16	0.45 × 0.42 × 0.32
No. of measured, independent and observed [*I* > 2σ(*I*)] reflections	8763, 2625, 2622	60,640, 12,299, 11,734	16,684, 3038, 2922	16,666, 3125, 3114
*R*_int_	0.016	0.025	0.021	0.025
(sin θ/λ)_max_ (Å^−1^)	0.618	0.619	0.618	0.618
*R*[*F*^2^ > 2σ(*F*^2^)], *wR*(*F*^2^), *S*	0.023, 0.063, 1.06	0.052, 0.128, 1.10	0.032, 0.084, 1.04	0.031, 0.083, 1.07
No. of parameters	233	925	229	234
No. of restraints	1	0	0	0
Δ_max_, Δ_min_ (e Å^−3^)	0.19, −0.12	2.56, −0.85	0.29, −0.24	0.42, −0.28
Absolute structure	Flack (1983)	–	–	–
Absolute structure parameter	0.14 (3)	–	–	–

**Table 4:** Hydrogen-bond geometry (Å, °) for **3a**.

*D*—H⋯*A*	*D*—H	H⋯*A*	*D*⋯*A*	*D*—H⋯*A*
N4—H4*A*⋯N2 ^i^	0.86 (3)	2.57 (2)	3.2842 (19)	141 (2)
N4—H4*B*⋯N42 ^ii^	0.93 (3)	2.24 (2)	3.0384 (18)	143 (2)

Symmetry codes: (^i^) −*x*, *y* − 1/2, −*z*; (^ii^) −*x*, *y* + 1/2, −*z*.

**Table 5:** Hydrogen bond geometry (Å, °) in **3b**.

*D*—H⋯*A*	*D*—H	H⋯*A*	*D*⋯*A*	*D*—H⋯*A*
N4*B*—H4*BA*⋯N2*A*	0.86 (3)	2.25 (3)	3.041 (3)	153 (3)
N4*D*—H4*DA*⋯O33*B*	0.87 (4)	2.49 (4)	3.125 (3)	130 (3)
N4*A*—H4*AB*⋯N2*B*	0.86 (3)	2.33 (3)	3.107 (3)	150 (3)

**Table 6:** Hydrogen bond geometry (Å, °) in **4a**.

*D*—H⋯*A*	*D*—H	H⋯*A*	*D*⋯*A*	*D*—H⋯*A*
N3—H3⋯O33 ^i^	0.862 (17)	2.278 (17)	3.1072 (13)	161.6 (17)

Symmetry codes: (^i^) *x −* 1, −*y* + 3/2, *z* − 1/2.

**Table 7:** Hydrogen-bond geometry (Å, °) in **4b**.

*D*—H⋯*A*	*D*—H	H⋯*A*	*D*⋯*A*	*D*—H⋯*A*
O1*W*—H1*W*⋯O4 ^i^	0.81 (2)	2.14 (2)	2.9372 (13)	169 (2)
N3—H3⋯O1*W*	0.88	2.39	3.1280 (16)	142

Symmetry codes: (^i^) *x*, *y* − 1, *z*.

## Data Availability

Data are contained within the article.

## Supplementary Materials

The following supporting information can be downloaded at: https://www.mdpi.com/article/10.3390/ma15093085/s1, Figure S1: Powder diffraction patterns for the studied compounds- experimental and theoretical calculated for single crystal structure, Scheme S1: CSD quests for similar molecular fragments. Tn refer to number (n) of atoms bonded, Table S1: Selected bonds lengths (Å) in the studied structures and the mean values found in similar compounds within Cambridge Structural Database [56]; “X“ denoted -NH_2_ in **3a**,**b** and =O in **4a**,**b**, respectively, Table S2: Selected torsion angles and dihedral angles (phy/phe) between planes defined by the pyridine and phenyl rings (°).
