# Supplementary material for: Differences in the Structure and Antimicrobial Activity of Hydrazones Derived from Methyl 4-Phenylpicolinimidate
Source: Materials (Basel). 2022 Apr 24;15(9):3085. doi: 10.3390/ma15093085 (PMC9103619; doi:10.3390/ma15093085)
Supplement: Supplementary file 1 [file materials-15-03085-s001.zip › materials-1673092-supplementary.pdf]

Article

Hydrazones Derived from Methyl 4-Phenylpicolinimidate: Differences in Structure and Antimicrobial Activity

Katarzyna Gobis [1,*], Małgorzata Szczesio [2], Andrzej Olczak [2], Izabela Korona-Głowniak [3], Ewa Augustynowicz-Kopeć [4], Ida Mazernt-Politowicz [2], Dagmara Ziembicka [1] and Marek L. Główka [2]

1 Department of Organic Chemistry, Medical University of Gdańsk, 107 Gen. Hallera Av., 80-438 Gdansk, Poland; katarzyna.gobis@gumed.edu.pl (K.G.); dagmara.ziembicka@gumed.edu.pl (D.Z.)
2 Institute of General and Ecological Chemistry, Faculty of Chemistry, Lodz University of Technology, Zeromskiego 116, 90-924 Lodz, Poland; malgorzata.szczesio@p.lodz.pl (M.S.); andrzej.olczak@p.lodz.pl (A.O.); marek.glowka@p.lodz.pl (M.L.G.); ida.mazerant-politowicz@dokt.p.lodz.pl (I.M.P.)
3 Chair and Department of Pharmaceutical Microbiology, Faculty of Pharmacy, Medical University of Lublin, 1 Chodźki Street, 20-093 Lublin, Poland; iza.glowniak@umlub.pl
4 Department of Microbiology, National Tuberculosis and Lung Diseases Research Institute, 26 Płocka Str., 01-138 Warsaw, Poland; e.kopec@igichp.edu.pl
* Correspondence: katarzyna.gobis@gumed.edu.pl; Tel.: +48-58-349-16-47

Supplementary Materials

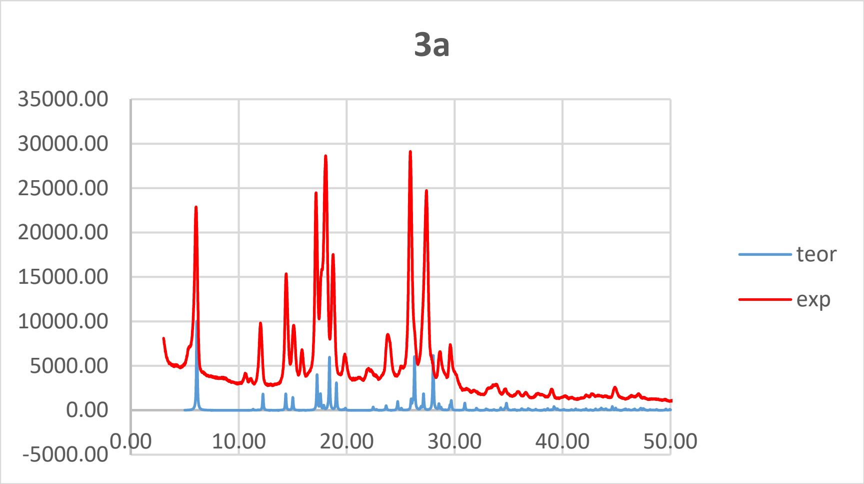

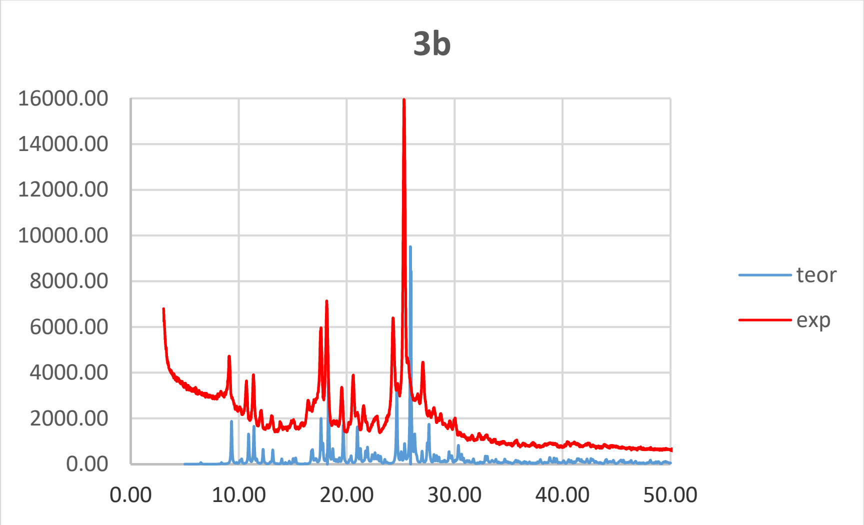

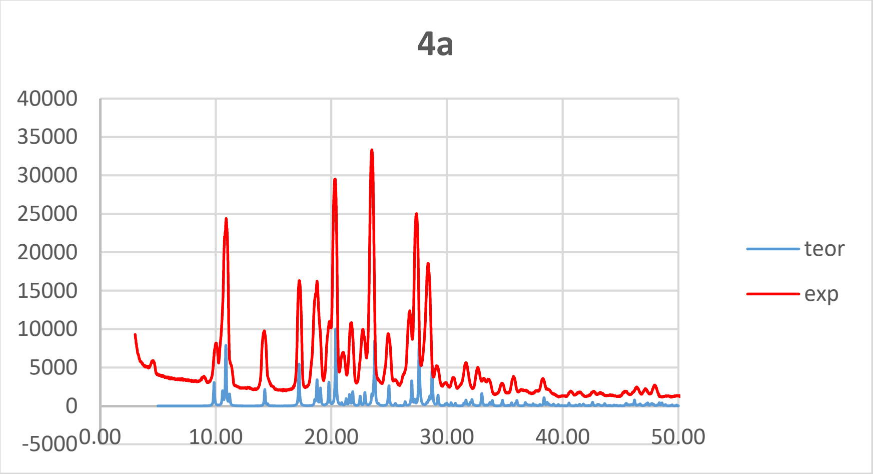

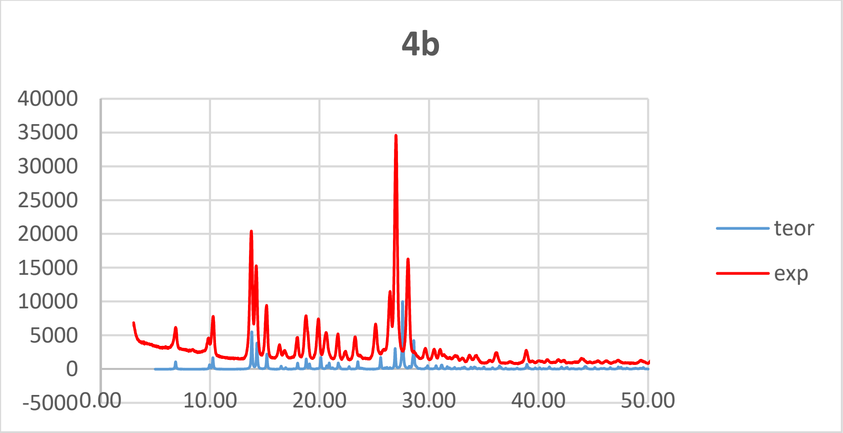

Figure S1. Powder diffraction patterns for the studied compounds- experimental and theoretical calculated for single crystal structure.

Table S1. Selected bonds lengths (Å) in the studied structures and the mean values found in similar compounds within Cambridge Structural Database [54]. CSD quests for molecular fragments are shown in Scheme A1. "X" denoted -NH_2 in **3a,b** and =O in **4a,b**, respectively.

Crystal Structure(s)	C1-C	C1-N2	N2-N3	N3-C4	C4-C41	C4-X4(N,O)
**3a**	1.440(3)	1.285(2)	1.390(2)	1.311(2)	1.491(2)	1.342(2)
**3b**	1.453(3)<br>1.452(3)<br>1.448(4)<br>1.441(4)	1.290(3)<br>1.292(3)<br>1.280(5)<br>1.293(4)	1.390(3)<br>1.392(3)<br>1.397(3)<br>1.391(3)	1.312(3)<br>1.304(3)<br>1.298(4)<br>1.313(3)	1.489(3)<br>1.496(3)<br>1.485(4)<br>1.482(4)	1.333(3)<br>1.335(3)<br>1.344(5)<br>1.343(4)
Mean for **3a,b**	**1.447(5)**	**1.288(3)**	**1.391(3)**	**1.310(5)**	**1.489(4)**	**1.339(3)**
Mean (CSD)	**1.464(4)**	**1.279(5)**	**1.402(4)**	**1.306(6)**	**1.489(3)**	**1.341(4)**
**4a**	1.445(2)	1.280(2)	1.368(2)	1.361(2)	1.502(2)	1.222(1)
**4b**	1.447(2)	1.282(2)	1.369(2)	1.355(2)	1.504(2)	1.226(2)
Mean for **4a,b**	**1.446(2)**	**1.281(2)**	**1.369(2)**	**1.358(2)**	**1.503(2)**	**1.224(2)**
Mean (CSD)	**1.459(8)**	**1.278(5)**	**1.375(5)**	**1.349(6)**	**1.500(5)**	**1.224(5)**

Table S2. Selected torsion angles and dihedral angles (phy/phe) between planes defined by the pyridine and phenyl rings (°).

Crystal structure	C-C1-N2-N3	C1-N2-N3-C4	N2-N3-C4-C41	N3-C4-C41-N42	pyr/phe
3a	-179.8(1)	-178.3(1)	178.9(1)	-179.8(1)	28.5(2)
3b	-1.2(4)<br>0.3(3)<br>2.8(4)<br>0.1(4)	-178.9(2)<br>-175.3(2)<br>-169.5(3)<br>-177.4(2)	-177.2(2)<br>-179.4(2)<br>-175.3(2)<br>-177.9(2)	170.3(2)<br>-172.7(2)<br>-177.2(3)<br>176.8(2)	24.8(2)<br>31.8(3)<br>31.9(3)<br>13.8(2)
4a	179.1(1)	-173.3(1)	179.2(1)	1.2(2)	37.5(2)
4b	-178.4(1)	-173.8(1)	177.1(1)	6.7(2)	28.6(2)

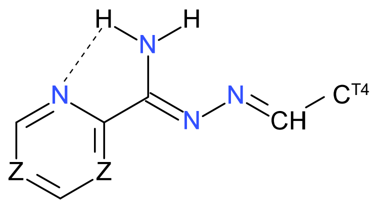

3a,3b, Z-CH or N
Found - 5 structures,
6 independent molecules

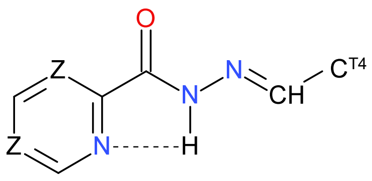

4a,4b, Z-CH or N
Found - 39 structures,
49 independent molecules

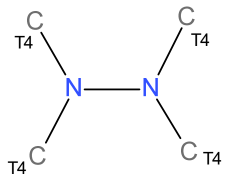

Found - 3 structures with N-N bond lenghsof about 1.50Å[Refcode: AZBNOW, RETHAZ, SOFQOT]

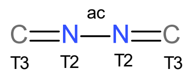

Found - 536 structures,
619 independent molecules
1.403(9) mean N-N
1.208(8) mean N=C

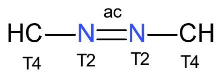

Found - 7 structures,
7 independent molecules
1.234(5) mean N=N

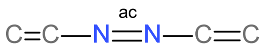

Found - 3 structures,
4 independent molecules
1.283(9) mean N=N
1.382(9) mean N-C

Scheme S1. CSD quests for similar molecular fragments. Tn refer to number (n) of atoms bonded.
